# Expression of Paramyxovirus V Proteins Promotes Replication and Spread of Hepatitis C Virus in Cultures of Primary Human Fetal Liver Cells

**DOI:** 10.1002/hep.24557

**Published:** 2011-12-02

**Authors:** Linda Andrus, Svetlana Marukian, Christopher T Jones, Maria Teresa Catanese, Timothy P Sheahan, John W Schoggins, Walter T Barry, Lynn B Dustin, Kartik Trehan, Alexander Ploss, Sangeeta N Bhatia, Charles M Rice

**Affiliations:** 1 Center for the Study of Hepatitis C, Laboratory of Virology and Infectious Diseases, The Rockefeller UniversityNew York, NY; 2 Division of Health Sciences and Technology, Department of Electrical Engineering and Computer Science, Howard Hughes Medical Institute, Massachusetts Institute of TechnologyCambridge, MA

## Abstract

Here we demonstrate that primary cultures of human fetal liver cells (HFLC) reliably support infection with laboratory strains of hepatitis C virus (HCV), although levels of virus replication vary significantly between different donor cell preparations and frequently decline in a manner suggestive of active viral clearance. To investigate possible contributions of the interferon (IFN) system to control HCV infection in HFLC, we exploited the well-characterized ability of paramyxovirus (PMV) V proteins to counteract both IFN induction and antiviral signaling. The V proteins of measles virus (MV) and parainfluenza virus 5 (PIV5) were introduced into HFLC using lentiviral vectors encoding a fluorescent reporter for visualization of HCV-infected cells. V protein-transduced HFLC supported enhanced (10 to 100-fold) levels of HCV infection relative to untransduced or control vector-transduced HFLC. Infection was assessed by measurement of virus-driven luciferase, by assays for infectious HCV and viral RNA, and by direct visualization of HCV-infected hepatocytes. Live cell imaging between 48 and 119 hours postinfection demonstrated little or no spread of infection in the absence of PMV V protein expression. In contrast, V protein-transduced HFLC showed numerous HCV infection events. V protein expression efficiently antagonized the HCV-inhibitory effects of added IFNs in HFLC. In addition, induction of the type III IFN, IL29, following acute HCV infection was inhibited in V protein-transduced cultures. *Conclusion*: These studies suggest that the cellular IFN response plays a significant role in limiting the spread of HCV infection in primary hepatocyte cultures. Strategies aimed at dampening this response may be key to further development of robust HCV culture systems, enabling studies of virus pathogenicity and the mechanisms by which HCV spreads in its natural host cell population. (Hepatology 2011;54:1901-1912)

Acute hepatitis C virus (HCV) infection in humans frequently progresses to chronicity,^1^ and virus persistence in the liver has been suggested to result, at least in part, from the ability of the virus to antagonize the interferon (IFN) system.^2^-^5^ Paradoxically, our ability to culture the virus for prolonged periods in differentiated primary hepatocytes *in vitro* has met with variable success.^6^-^11^

Use of the hepatoma line Huh-7 and its derivatives and adaptation of viral genomes to propagation in these cells has made possible the generation of high titer stocks of cell culture-derived HCV (HCVcc),^12^, ^13^ enabling the identification of cellular factors required for virus entry and replication.^14^-^18^ It has become apparent, however, that hepatoma lines may not fully recapitulate all aspects of HCV replication in the liver, and that host responses play an important part in determination of viral persistence or clearance. For example, nucleotide polymorphisms in or near the gene for the type III IFN, IL-28B, were recently shown to be predictive of resolution of acute HCV infection, or favorable response to IFN-alpha/ribavirin therapy in infected patients.^19^ The profound effect of these host polymorphisms may suggest a weak point in HCV's ability to evade the innate or adaptive immune response.

In comparison to hepatoma lines, complex cultures of primary human hepatocytes from genetically diverse donors may provide a more informative environment for studying the virus life cycle and cellular mechanisms that may operate to limit virus spread. In the present study we examined the efficiency of HCVcc replication in primary human fetal liver cell cultures (HFLC). To investigate the possible role of the innate immune system in controlling productive HCV infection in these cultures we exploited the well-characterized ability of paramyxovirus (PMV) V proteins to counteract both IFN induction^20^ and antiviral signaling mediated by binding of the IFN receptor.^21^

All PMV genomes encode a unique open reading frame termed V. Although diverse in overall amino acid sequence (only ≍50% sequence identity between PMV family members) all V proteins share a conserved cysteine-rich C-terminus that interacts with the RNA helicase domain of the pattern recognition receptors (PRR) MDA5 and LGP2.^22^, ^23^*In vitro*, V proteins have been shown to block induction of type I IFN in response to stimuli that activate the MDA5 pathway.^24^, ^25^ Evidence to date indicates that V proteins do not engage or antagonize the related RNA helicase RIG-I.^20^, ^22^

More extensively characterized is the ability of V proteins to potently inhibit cytokine signaling pathways by targeting STAT (signal transducer and activation of transcription).^21^ Receptor engagement by type I and type III IFNs results in the dimerization of STAT1 and STAT2 and assembly of the transcription complex ISGF3 that mediates expression of antiviral genes.^21^, ^26^, ^27^ The STAT molecules targeted by V proteins, and the mechanisms by which STAT signaling is inhibited, vary greatly between different PMV family members. The V protein of parainfluenza virus 5 (PIV5) targets STAT1 for proteosomal degradation by recruitment and assembly of components of the cellular E3 ubiquitin ligase machinery.^28^, ^29^ The V protein of measles virus (MV) targets both STAT1 and STAT2 and prevents their nuclear translocation in response to IFN receptor binding.^30^, ^31^

We introduced the V proteins of PIV5 and MV into cultured HFLC using bi-cistronic lentiviral vectors encoding a fluorescent reporter that permits direct visualization of HCV-infected cells.^32^ Our results show that V protein expression significantly enhances productive infection of HFLC with HCVcc, protects these cultures against the HCV-inhibitory effects of added type I and type III IFNs and antagonizes the induction of the type III IFN IL-29 in response to HCV infection. We also show by live-cell imaging that V protein expression dramatically enhances the early spread of HCV infection in these cultures.

## Patients and Methods

### Human Subjects

All protocols involving human tissue were reviewed and exempted by the Rockefeller University Institutional Review Board.

### Isolation and Culture of HFLC

Deidentified fetal livers (16-24 weeks gestation) were procured through Advanced Bioscience Resources (ABR; Alameda, CA) or the Human Fetal Tissue Repository of the Albert Einstein College of Medicine (AECOM; New York, NY). Livers received on ice were washed with hepatocyte wash buffer (HWB) consisting of Williams' E Medium (WEM) plus 10 mM HEPES, 50 μg/mL gentamicin, 100 U/mL penicillin, and 100 μg/mL streptomycin (Invitrogen). Tissue was minced then resuspended in 20-40 mL warm digestion buffer consisting of Hanks Balanced Salt Solution plus 40 mM HEPES, 3.26 mM CaCl_2_, 2 U/mL DNase I Grade II (Roche), and 0.2% Collagenase type IV (Sigma). Tissue was digested for 30 minutes at 37°C, then diluted 1:1 with HWB and gently pushed through 70 μm cell-strainers (BD Biosciences). The suspension was centrifuged at 100*g* for 3 minutes and the cell pellet containing large hepatocytes was washed twice by resuspension in 50 mL HWB and centrifugation at 100*g* for 4 minutes. Hepatocytes were enriched by 1*g* sedimentation in 25 mL HWB for 1 hour at room temperature, followed by additional washing. In some experiments hepatocytes were further enriched by centrifugation through lymphocyte separation medium (Cellgro, Manassas, VA) as described.^33^ Hepatocyte yields ranged from 0.5 to 4 × 10^7^ cells per tissue and cells were generally >80% viable as assessed by Trypan blue exclusion and collagen attachment. Hepatocytes were plated at ≍1 × 10^5^/cm^2^ on 24- or 48-well collagen I-coated plates (BD Biosciences) in WEM containing 10% fetal bovine serum (FBS) (Omega Scientific, Tarzana, CA), 2 mM L-glutamine (Invitrogen), 1X ITS Plus (BD Biosciences) and antibiotics. After overnight incubation, adherent cells were washed with WEM, then maintained in Hepatocyte Defined Medium (HDM; BD Biosciences) plus L-glutamine and antibiotics. The culture medium was aspirated and replaced every 2 days.

### Lentiviral Vectors

V proteins and control protein firefly luciferase (Fluc) were expressed from a hybrid albumin promoter in a bi-cistronic lentiviral vector^34^ modified to express the HCV-dependent fluorescence relocalization (HDFR) cassette TagRFP-NLS-IPS.^32^ In this cassette the fluorescent reporter TagRFP is fused to both a nuclear localization sequence (NLS) and the transmembrane domain of the mitochondrially tethered adapter protein IPS-1.^32^ Following HCV infection of HDFR-expressing cells, cleavage of IPS-1 by the viral NS3-4A protease^2^ leads to migration of the fluorescent reporter from mitochondria to the nucleus, enabling visualization of HCV-infected cells.^32^ Vector construction and source of protein-coding sequences are detailed in the Supporting Information and Supporting Fig. S1A.

### Transduction with Lentiviral Pseudoparticles (PP)

PP were prepared by cotransfection of 293T cells with lentiviral and packaging plasmids as described.^15^, ^32^ HFLC were transduced 1-3 days postplating by incubation for 3-6 hours with PP stocks diluted 1:3 in HDM plus 20 mM HEPES and 4 μg/mL polybrene, then washed and fed with HDM.

### HCVcc Inocula

HCVcc inocula used were the Gaussia luciferase reporter virus Jc1FLAG2 (p7-nsGluc2A)^35^ (JC1G) and J6JFH Clone 2,^36^ which lacks a reporter but replicates to higher titer in Huh-7.5 cells. Virus stocks were prepared by electroporation of *in vitro* transcribed RNA into Huh-7.5 cells as described^35^ and virus was collected in serum-free medium, or medium containing 1.5 or 10% FBS. For some experiments virus stocks were concentrated using Amicon Ultracel-100K filters (Millipore). Infectious titers of HCVcc inocula were determined by titration on Huh-7.5 cells as described^12^ and are expressed as 50% tissue culture infectious doses (TCID_50_). HFLC were infected for 3 to 6 hours with HCVcc diluted in HDM, then washed and fed with HDM.

### Detection of HCV RNA

Total RNA was extracted from washed HFLC using RNEasy Kits (Qiagen). HCV RNA was detected by quantitative RT-PCR using the Eragen MultiCode-RTx method (Eragen Biosciences, Madison, WI) and primers directed to the 5′ untranslated region of the HCV genome. Quantitation of HCV RNA copy number was achieved using a synthetic RNA standard (Apath, Brooklyn, NY).

### Cytokines and Drugs

Recombinant human cytokines, IFN-beta (IFN-β), IL-28A, and IL-29, were from Peprotech (Rocky Hill, NJ). The HCV NS5B polymerase inhibitor 2′ C-methyl adenosine (2′CMA) was the gift of D. Olsen and S. Carroll (Merck Research Laboratories, West Point, PA).

### Immunofluorescence Analysis (IFA), Immunoblotting, and Enzyme-Linked Immunosorbent Assay (ELISA)

IFA^9^ and immunoblotting^14^ were carried out as described. Antibodies and reagents are detailed in the Supporting Information. Human IL-29 was detected using ELISA kits from eBioscience (San Diego, CA). ELISA for human albumin is described in the Supporting Information.

### Time-Lapse Live Cell Imaging

HFLC were plated on collagen-coated optical dishes and maintained in HDM. Images were acquired on a Zeiss Axiovert-200 inverted microscope equipped with an environmental chamber. Visualization of Tag-RFP in transduced HFLC was achieved by laser excitation and emission as described.^32^

## Results

### Cultured HFLC Are Long-Lived and Express HCV Entry Factors

**Fig. 1:**
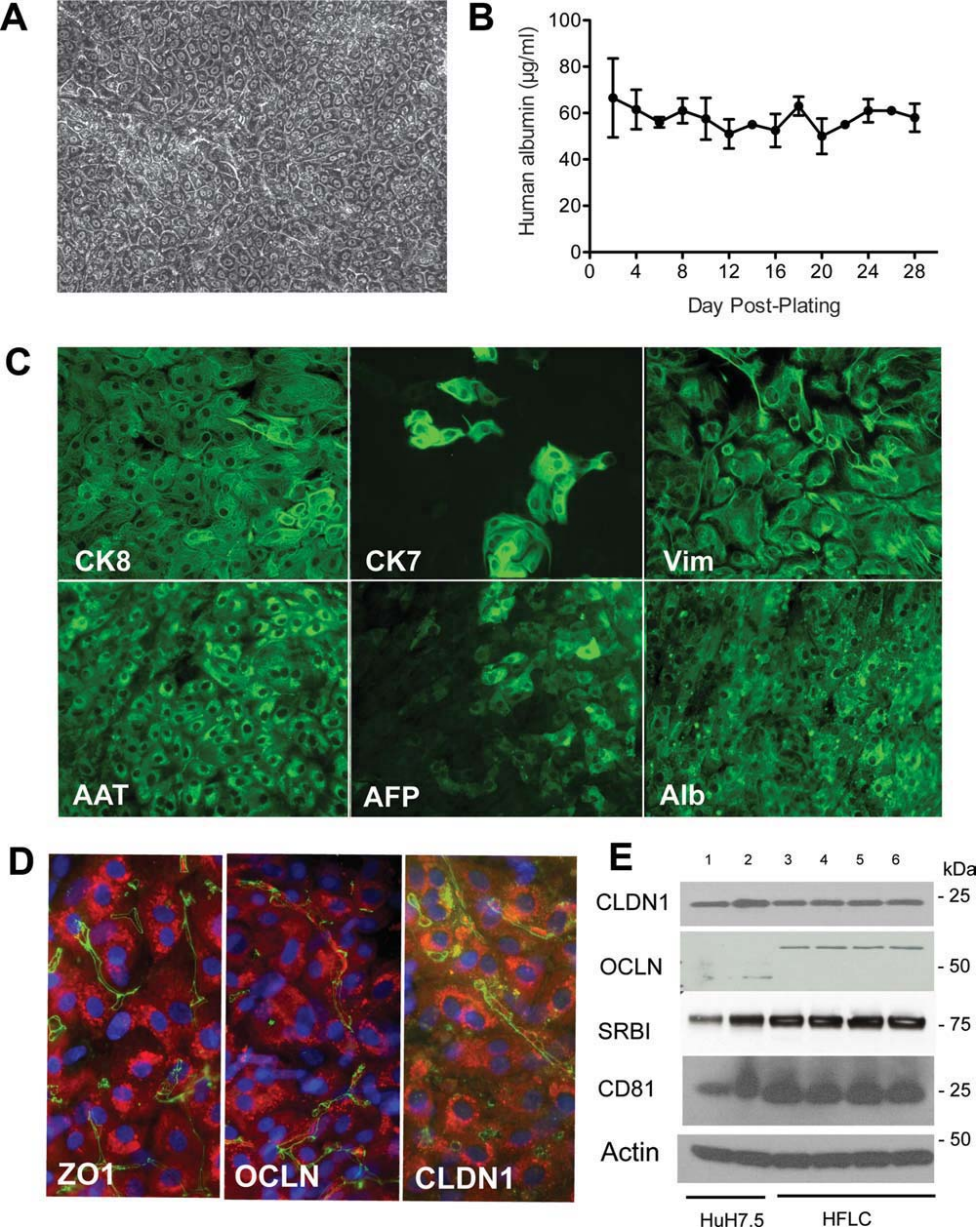
Cultured HFLC are long-lived, express markers of differentiated hepatocytes, and are positive for the expression of HCV entry factors. (A) Phase contrast microscopy of HFLC 2 weeks postplating. (B) Albumin secretion by HFLC during 1 month of culture (mean and SD of three cultures). (C) Antibody staining for cytokeratin 8 (CK8), cytokeratin 7 (CK7), vimentin (Vim), alpha-1 antitrypsin (AAT), alpha fetoprotein (AFP), and albumin (Alb) in cultured HFLC 1 week postplating. Bound antibody was detected with AlexaFluor (AF)-488-conjugated antibody to immunoglobulins. (D) AF-488 (green) detection of bound antibodies to the tight junction protein zona occludens 1 (ZO1) and the HCV entry factors occludin (OCLN) and claudin 1 (CLDN1) in cultured HFLC 1 week postplating. Hepatocyte staining for AAT (red) was detected with AF-594-conjugated antibody to goat IgG. Nuclei are counterstained with DAPI (blue). (E) Western blot for HCV entry factors in lysates of Huh-7.5 cells and HFLC 1 week postplating: Lane 1, 15 μg Huh-7.5 lysate; Lane 2, 30 μg Huh-7.5; Lane 3, 30 μg HFLC. We also examined entry factor expression in HFLC transduced with each of the three lentiviral vectors described below. Lanes 4-6 correspond to 30 μg lysate from HFLC transduced with pseudoparticles encoding: Lane 4, firefly luciferase; Lane 5, parainfluenza virus 5 V protein; Lane 6, measles virus V protein. Lysates were prepared 6 days posttransduction. SRBI, scavenger receptor BI. Note that HFLC express the ≍60 kDa form of OCLN that is found in adult human liver,^37^ and absent or weakly expressed in Huh-7.5 cells.

Like other investigators, we found human fetal liver-derived hepatocytes to be long-lived compared with cultured adult human hepatocytes.^6^, ^7^, ^9^ Figure 1A shows phase contrast morphology of cultured HFLC. Hepatocytes formed a tightly packed monolayer with refractive cell margins indicative of the formation of canaliculi. Hepatocytes retained this morphology and continued to secrete albumin for at least 1 month in culture (Fig. 1B).

At 16 to 24 weeks of gestation, human fetal liver is considered to contain a mix of bi-potential hepatoblasts (capable of forming both hepatocytes and cholangiocytes) and their more committed progeny. Figure 1C shows IFA for the liver-derived proteins alpha-1 antitrypsin (AAT), alpha fetoprotein (AFP), and albumin, and for intermediate filament proteins in cultured HFLC. Hepatocytes stained uniformly positive for albumin and AAT and unevenly for AFP. CK8 expression was seen in both hepatocytes and cholangiocytes; the latter were visible as frequent clusters of CK7-positive cells within the hepatocyte monolayer. Unlike adult hepatocytes, fetal liver epithelial cells commonly coexpress mesenchymal markers such as vimentin in conjunction with hepatocyte markers.^38^ As shown in Fig. 1C, cultured HFLC showed strong staining for vimentin in cells with hepatocyte morphology and in cells with fibroblast morphology.

CLDN1 and OCLN, two of the four known entry factors for HCV, are tight junction proteins expressed preferentially at the canalicular domain in adult liver.^16^, ^17^ In cultured HFLC, CLDN1, OCLN, and the zona occludens marker ZO1 showed similar canalicular staining patterns (Fig. 1D). Immunoblot analysis of all four HCV entry factors (CLDN1, OCLN, SRBI, and CD81) in HFLC showed levels of expression comparable to those of the HCV-susceptible hepatoma Huh-7.5 (Fig. 1E). Taken together, these results show that cultured HFLC, although immature in phenotype, express all of the entry factors required for HCVcc infection.

### Cultured HFLC Are Susceptible to Infection with HCVcc

**Fig. 2:**
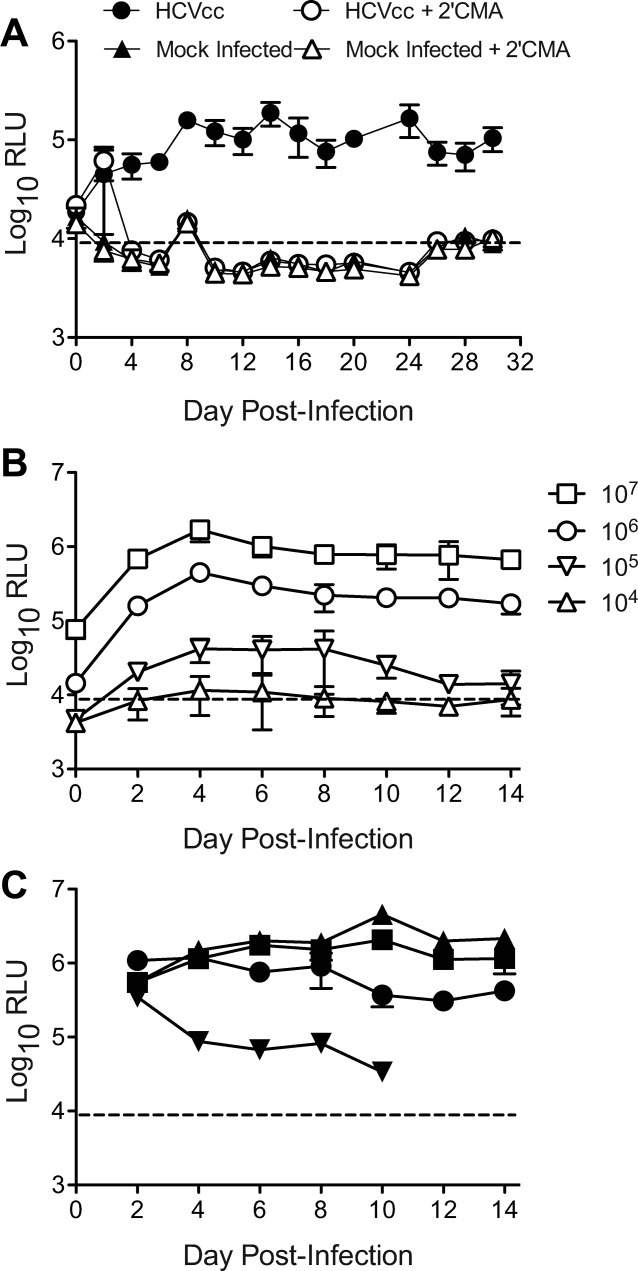
Cultured HFLC are susceptible to infection with HCVcc. (A) Gaussia luciferase secretion by HFLC (ABR-15168, 23 weeks gestation) following infection with the reporter virus JC1G in the presence or absence of the polymerase inhibitor 2′CMA (2.2 μm). Mock-infected cells were incubated with culture medium alone. Polymerase inhibitor treatment was discontinued after day 6 of culture. Secreted luciferase was measured at each medium change (mean and SD of three cultures). RLU, relative light units. The dashed line indicates mean plus 2 SD of RLU detected in culture medium alone. (B) Doses of HCVcc required for establishment of detectable infection. HFLC (AECOM-052810, gestational age not available) were incubated with the indicated tissue culture infectious doses (TCID_50_) of JC1G for 5 hours, washed, and refed with HDM. Secreted luciferase was measured at each medium change (mean and SD of three cultures). (C) JC1G infection of four different HFLC preparations with ≍10^5^ TCID_50_ of a single virus stock that was prepared in medium containing 10% v/v FBS.

We first tested the ability of cultured HFLC to support productive infection with HCVcc using the reporter virus JC1G, with Gaussia luciferase secretion as the readout for infection. Figure 2A shows that HFLC were susceptible to HCVcc infection and secreted low but measurable levels of luciferase, which persisted for at least 4 weeks of culture. Luciferase secretion was reduced by addition of the polymerase inhibitor 2′CMA during the first 6 days of culture, demonstrating the signal to be dependent on HCV RNA replication.

Figure 2C shows the results of an experiment in which HFLC were infected with graded doses of JC1G ranging from 10^4^-10^7^ TCID_50_. Establishment of detectable infection required virus doses >10^5^ TCID_50_ per culture (i.e., ≍1 TCID_50_ per seeded hepatocyte). Intriguingly, at all virus doses tested levels of secreted luciferase did not increase over time postinfection, but rather persisted at levels similar to those achieved during the first 2 days after virus inoculation (Fig. 2B). We also observed significant donor-to-donor variability in both the magnitude and duration of HCV replication between HFLC infected with equivalent doses of the same virus stock (Fig. 2C). In some experiments reporter virus replication declined in a manner suggestive of active viral clearance (Fig. 2C, and see below).

These results are similar to those previously obtained with HCVcc infection of micropatterned adult human hepatocytes.^9^ They are in marked contrast to the exponential virus amplification obtained following HCVcc infection of Huh-7.5 cells.^32^ We hypothesized that, despite the documented ability of HCV to counteract innate antiviral sensing and signaling pathways,^2^-^5^ spread of HCV infection in primary HFLC may be limited by the IFN system. To investigate this hypothesis we exploited the ability of PMV V proteins to antagonize both IFN induction and IFN signaling by way of STAT.

### V Protein Expression Promotes Productive HCVcc Infection in HFLC

We selected two PMV V proteins that differ in their STAT targeting specificity and mechanism of STAT inhibition. Expression of PIV5 and MV V proteins, and control protein Fluc, in HFLC was achieved by transduction with lentiviral PP encoding the HDFR cassette, which permits visualization of HCV-infected cells by nuclear translocation of RFP.^32^ Vector constructs and characterization of V protein expression are described in the Supporting Information and Supporting Fig. S1. Protein expression was found to be efficient in Huh-7 cells and hepatocytes (Supporting Fig. S1B-D), and vector transduction was found not to compromise HCV replication in HFLC (Supporting Fig. S1E).

**Fig. 3:**
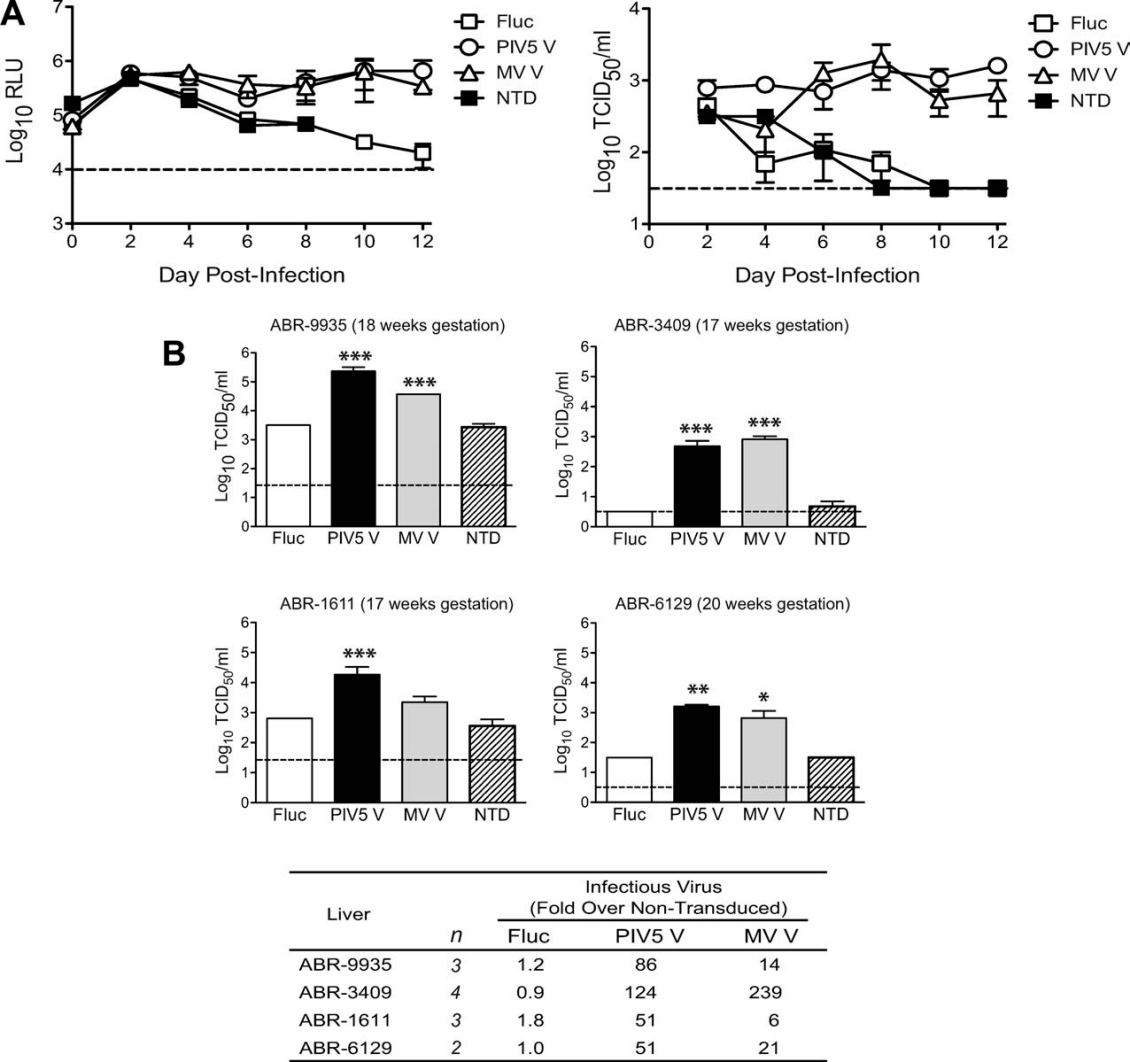
PMV V protein expression enhances productive infection with JC1G in cultured HFLC. (A) One day postplating, HFLC (ABR-5591, 17 weeks gestation) were transduced with lentiviral vectors encoding firefly luciferase (Fluc), parainfluenza virus 5 V protein (PIV5 V), or measles virus V protein (MV V), or left nontransduced (NTD). Five days later cells were infected with 1 × 10^6^ tissue culture infectious doses (TCID_50_) JC1G reporter virus. Washed cells were cultured in HDM and the medium was removed and replaced every 2 days. Harvested supernatants were assayed for luciferase (left panel) and for levels of infectious virus by titration on Huh-7.5 cells (right panel). RLU, relative light units. Values show mean and SD of three cultures. The dashed line indicates the lower limit of detection for each assay. (B) Levels of infectious virus recovered from cell supernatants 2 weeks post-JC1G infection of four different HFLC preparations. Graphs show mean and SD of 2-4 cultures. Log-transformed TCID_50_ values within each experiment were analyzed by 1-way analysis of variance (ANOVA) with Bonferroni multiple comparison posttest. Values that differ significantly from both nontransduced (NTD) and Fluc-transduced cultures are indicated: *P < 0.05; **P < 0.01; ***P < 0.001. Fold differences relative to nontransduced cultures are summarized below the graphs.

HFLC were either left untransduced or transduced with PP 1-3 days postplating, then infected with JC1G 4-6 days later. Figure 3A shows the effect of V protein transduction in one experiment, using both luciferase secretion (left panel) and infectious virus production (right panel) as the readouts for infection. Both luciferase secretion and virus release declined steadily between days 2 and 12 postinfection in control cultures. In contrast, cultures transduced with either PIV5 or MV V protein showed persistence of luciferase and maintenance of virus production over the same time period. Results from four independent infection experiments are shown in Fig. 3B. In each experiment, levels of infectious HCV recovered from HFLC supernatants at 2 weeks postinoculation were ≍1-2 logs higher in V protein-transduced cultures than control cultures. These results suggest that V protein expression serves to enhance productive infection, or “rescue” an abortive infection with HCVcc in these primary cells. Similar results were obtained in micropatterned cultures of adult human hepatocytes, indicating that the enhancing effects of V proteins on HCV replication are not limited to fetal hepatocytes (see Supporting Information and Supporting Fig. S2).

### Direct Visualization of HCVcc Infection in Cultured HFLC

**Fig. 4:**
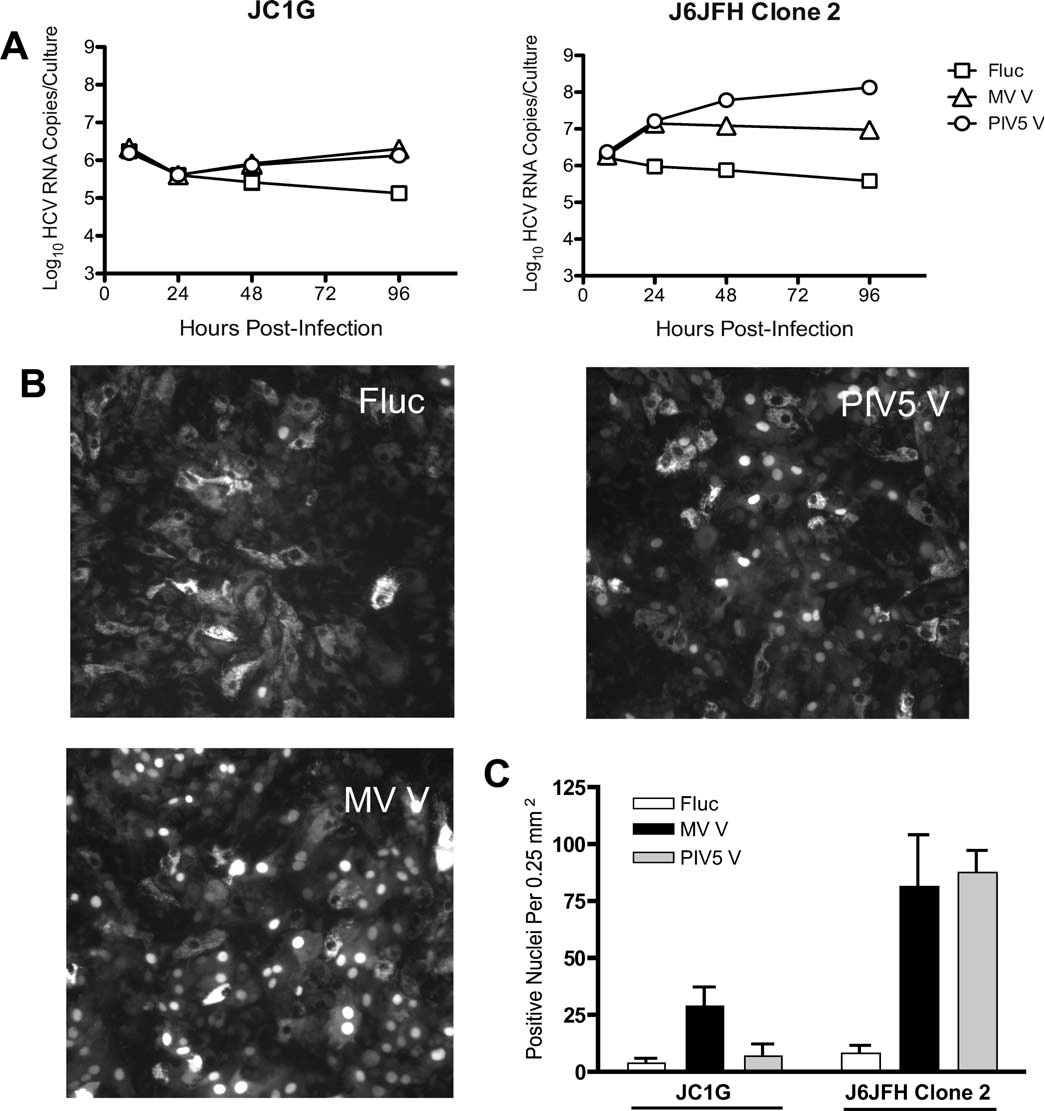
J6JFH Clone 2 replicates to higher levels in HFLC than JC1G, and replication is enhanced by V protein expression. Four days posttransduction with lentiviral vectors, HFLC (AECOM-063010; gestational age not available) were incubated with 1 × 10^7^ TCID_50_ JC1G or J6JFH Clone 2 for 6 hours, washed three times, and cultured in HDM. (A) Levels of cell-associated HCV RNA detected during the first 4 days postinfection (mean and SD of 2 assays on 2 cultures). (B) Representative images for HFLC infected with J6JFH Clone 2. (C) Numbers of RFP-positive nuclei per microscope field on day 7 postinfection (mean and SD of six fields from two cultures).

We compared the effects of V protein expression on replication of both JC1G and J6JFH Clone 2, which replicates to comparatively higher titers on Huh-7.5 cells.^36^ Figure 4A shows levels of cell-associated HCV RNA detectable during the first 96 hours postinfection with an equivalent dose of each virus. In Fluc-transduced HFLC, levels of RNA for both viruses declined slightly between 8 and 96 hours postinfection. In V protein-transduced HFLC, HCV RNA levels increased over the same time period, with levels of J6JFH Clone 2 RNA increasing about one log during the first 24 hours. Figure 4C shows a comparison of these two viruses using RFP nuclear translocation^32^ as the readout for infection 7 days postinoculation. Nuclear translocation events were more readily quantifiable for J6JFH Clone 2, particularly in the context of V protein expression (Fig. 4B,C).

**Fig. 5:**
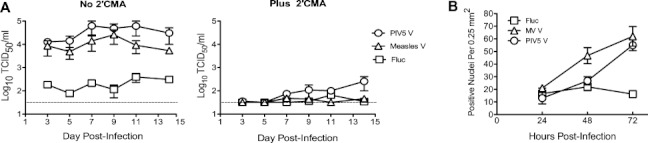
Kinetics of J6JFH Clone 2 replication in cultured HFLC. (A) Five days posttransduction, HFLC (AECOM-031010, gestational age unavailable) were incubated with 1 × 10^7^ TCID5_0_ J6JFH Clone 2, washed three times, and refed with HDM with or without of 2.2 μm 2′CMA. Cells were washed again and refed 24 hours postinfection. Treatment with 2′CMA was discontinued on day 7. Infectious virus in culture supernatants was determined by titration on Huh-7.5 cells. Values are mean and SD of four cultures. (B) Kinetics of appearance of RFP-positive nuclei during the first 3 days of culture following infection with J6JFH Clone 2 (ABR-8338; 20 weeks gestation). Values are mean and SD of nuclei counts from four fields of two cultures.

As with JC1G, V protein expression enhanced J6JFH Clone 2 replication in HFLC, permitting sustained, 2′CMA-sensitive production of high titers of infectious virus for 14 days (Fig. 5A). Scoring for RFP translocation events during the first 72 hours postinfection revealed a steady increase in the numbers of HCV-infected cells in V protein-transduced HFLC, but not in Fluc-transduced cultures (Fig. 5B).

**Fig. 6:**
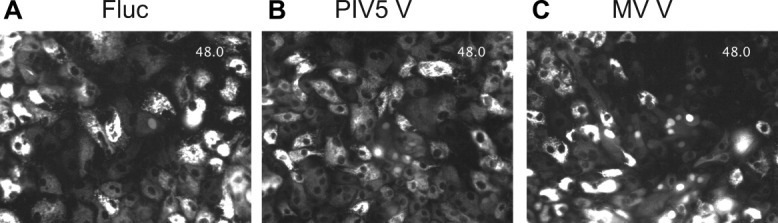
Live-cell imaging of J6JFH Clone 2 infection in PMV V protein- or Fluc-transduced HFLC. Five days posttransduction with lentiviral vectors, HFLC (ABR-3676; 19 weeks gestation) were infected with 1 × 10^7^ TCID_50_ J6JFH Clone 2. Washed cells were cultured in HDM for 48 hours prior to initiation of imaging. For each culture condition, 10 fields containing at least one cell with nuclear translocation of RFP were selected to monitor possible spread of infection. The figure shows the first frame of representative fields of cells transduced with (A). Firefly luciferase (Fluc). (B) Parainfluenza virus 5 V protein (PIV5 V) or (C) measles virus V protein (MV V). Full videos for each field are shown in Fig. 6A_Fluc.avi, Fig. 6B_PIV5 V.avi, and Fig. 6C_MV V.avi and are viewable in the online version of the manuscript. The time stamp indicates elapsed time in hours beginning at 48 hours postinfection.

The latter finding prompted us to carry out time-lapse live cell imaging to monitor the appearance of new RFP translocation events after HCVcc infection (Fig. 6). The results showed little or no spread of infection in Fluc-transduced cultures at 48-119 hours postinfection, whereas V protein-transduced cultures showed numerous new translocation events. Representative examples of 10 independent fields are shown for each culture condition (Fig. 6). Video files are viewable in the online version of the manuscript.

These results suggest that a major impact of V proteins in HFLC is to promote virus spread during the early stages of infection. V protein expression did not measurably affect levels of HCV entry factors in HFLC (Fig. 1E), or entry of HCV-enveloped PP (Supporting Fig. S3), suggesting that their enhancing effect on infectious HCV spread is not mediated at the level of virus entry. We next tested which of the V proteins' known anti-IFN functions (i.e., inhibition of STAT signaling and inhibition of IFN induction) were active in HFLC.

### V Proteins Counteract the HCV-Inhibitory Effects of Added IFNs in HFLC

**Fig. 7:**
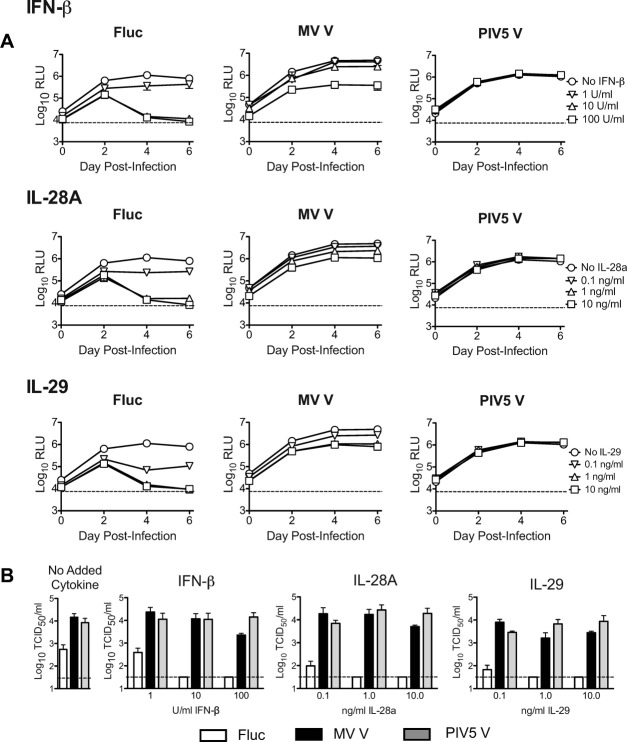
V protein expression counteracts the HCV-inhibitory effects of added type I and type III interferons in HFLC. Four days posttransduction with lentiviral vectors, HFLC (ABR-1108; 19 weeks gestation) were incubated with 1 × 10^6^ TCID_50_ per well JC1G reporter virus for 5 hours. Cells were washed three times then refed with HDM containing serial 10-fold dilutions of IFN-β, IL-28A, or IL-29. The culture medium was aspirated and replaced every 2 days. Cytokine treatment was discontinued after the second day of culture. (A) JC1G reporter virus-derived luciferase activity detected in culture supernatants during the first 6 days of culture (mean and SD of two cultures). RLU, relative light units. (B) Levels of infectious HCV detected in cell culture supernatants on day 14 of culture (mean and SD of two assays on two cultures). TCID_50_, tissue culture infectious doses; Fluc, firefly luciferase; PIV5 V, parainfluenza virus 5 V protein; MV V, measles virus V protein.

Although they bind to distinct membrane receptors, both type I and type III IFNs induce antiviral signaling by way of activation of STAT1 and STAT2.^26^, ^27^ We tested the ability of V proteins to counteract the HCV-inhibitory effects of IFN-β and type III IFNs, IL-28A, and IL-29 using the JC1G reporter virus to measure productive infection of HFLC (Fig. 7).

IFNs were added to the culture medium immediately after HCVcc infection and withdrawn after the second day of culture. In control-vector-transduced HFLC, all three IFNs inhibited HCV replication in a dose-dependent manner, with maximal inhibition occurring with 100 U/mL IFN-β, 100 ng/mL IL-28A, and 10 or 100 ng/mL IL-29 (Fig. 7A). With each dose of each IFN the level of HCV replication in HFLC transduced with PIV V protein was equivalent to that of cells cultured in the absence of added cytokine, indicating potent antagonism of STAT signaling and confirming the efficiency of lentiviral transduction in these cultures. HFLC transduced with MV V protein were likewise protected, and for most IFN doses, luciferase levels were equivalent to or greater than those of control cells cultured without added cytokine (Fig. 7A).

To confirm that the 2-day IFN dosing schedule used was sufficient to reduce productive HCV infection, we measured levels of infectious virus in HFLC supernatants after 14 days of culture (i.e., 12 days after removal of added IFN) (Fig. 7B). Infectious virus was recovered from supernatants of V protein-transduced, but not Fluc-transduced HFLC, treated with the maximally inhibitory doses of each IFN defined above. In addition, levels of HCV produced by HFLC cultured *without* added cytokine were significantly higher for V protein-transduced cells (27-fold for MV V, and 15-fold for SV5) than control cells (Fig. 7B, left panel). These data suggest that V protein expression counteracts the inhibitory effects of added IFN and also antagonizes endogenous cytokines generated during the course of infection.

### V Protein Expression Inhibits IL-29 Protein Induction in HCVcc-Infected HFLC

In the accompanying article we report that acute infection of HFLC with J6JFH Clone 2 results in the secretion of IL-29 into the culture supernatant and induction of mRNAs for IL-29 and IL-28B.^39^ Detectable induction of type III IFNs was less frequent in HFLC infected with the JC1G reporter virus, possibly reflecting the superior replication efficiency of J6JFH Clone 2 relative to JC1G.

**Fig. 8:**
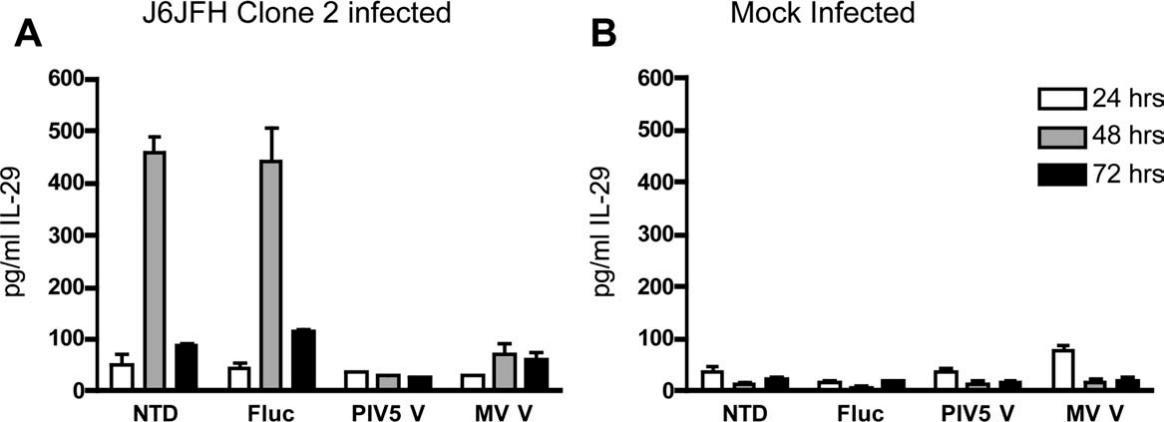
V protein expression inhibits IL-29 protein induction following HCV infection. Four days posttransduction with lentiviral vectors, cultures of gradient-enriched HFLC (ABR-8338; 20 weeks gestation) were incubated for 5 hours with (A) 1 × 10^6^ TCID_50_ per well J6JFH Clone 2, or (B) with an equivalent dilution of supernatant from mock-electroporated Huh-7.5 cells. Cells were washed three times then refed with HDM. The culture medium was removed and replaced daily and supernatants were assayed for IL-29 by ELISA (mean and SD of two cultures). Comparable results were obtained in two other experiments. NTD, no transduction; Fluc, firefly luciferase; PIV5 V, parainfluenza virus 5 V protein; MV V, measles virus V protein.

In light of these results, we determined whether PMV V protein expression would modulate IL-29 induction after acute infection of HFLC with J6JFH Clone 2. As shown in Fig. 8, both untransduced and control transduced HFLC showed a peak of IL-29 production at 48 hours postinfection with HCVcc, which declined by 72 hours. No detectable IL-29 production was seen in mock-infected HFLC (assay cutoff 50 ng/mL). In HFLC transduced with either PIV5 or MV V proteins, little or no IL-29 induction was seen at any timepoint postinfection with HCVcc.

## Discussion

We have shown that primary cultures of HFLC reliably support productive infection with HCVcc. Virus replication was sensitive to inhibition by the polymerase inhibitor 2′CMA (Figs. 2A, 5A), and to added IFNs (Fig. 7). Levels of virus replication varied significantly between different donor cell preparations and frequently declined in a manner suggestive of active viral clearance (Figs. 2, 3). At present, we do not know whether this variability is due to differences in cellular composition or state of differentiation of hepatocytes within these cultures or to genetic differences between tissue donors. Studies to date have not revealed an association with IL28B genotype.^39^

V protein-transduced HFLC supported significantly enhanced (≍10 to 100-fold) levels of HCV infection relative to untransduced or control vector-transduced HFLC. Infection was assessed by measurement of virus-driven luciferase, by assays for infectious HCV and viral RNA, and by direct visualization of HCV-infected hepatocytes (Figs. 3-5). Similar results were obtained with micropatterned cocultures of adult human hepatocytes^11^ (Supporting Fig. S2).

Time-lapse live cell imaging of HFLC 48-119 hours postinfection demonstrated little or no spread of infection in the absence of V protein expression. In contrast, V protein-transduced HFLC showed numerous HCV infection events (Fig. 6). To our knowledge, this is the first report of visualization of HCV spread in primary cells. During the course of live cell imaging we observed considerable turnover of HCV-infected cells. Cell turnover could indicate a direct cytopathic effect of HCV infection in primary hepatocytes. However, more comprehensive studies will be required to determine the survival time of HCV-infected cells relative to uninfected hepatocytes in these cultures.

V proteins did not measurably affect levels of expression of HCV entry factors in HFLC (Fig. 1), nor did they function to promote virus entry as assessed by experiments using HCV-enveloped pseudoparticles (Supporting Fig. S3), or measurements of cell-associated HCV RNA 8 hours postinfection (Fig. 4A). Taken together, these results suggest that V proteins exert their effect by mechanisms independent of an effect on virus entry. Consistent with their known role in counteracting innate immunity during PMV infection, we found V protein expression to efficiently antagonize the HCV-inhibitory effects of added IFNs in HFLC (Fig. 7).

Interestingly, induction of the type III IFN, IL-29, that follows acute HCVcc infection of HFLC^39^ was inhibited in V protein-transduced cultures (Fig. 8). Studies conducted predominantly in hepatoma lines have defined RIG-I as the primary PRR required for IFN induction after HCV infection.^5^ Because V proteins do not engage RIG-I, but do bind helicases MDA5 and LGP2,^20^, ^22^ our results may suggest that recognition of HCV RNA occurs differently in primary HFLC cultures, and that infection of these cells leads to the generation of RNA species that are capable of directly activating the MDA5 pathway. Alternatively, they may reflect V protein antagonism of the PRR up-regulation that has been shown to accompany IFN stimulation.^40^ Previous work from our laboratory has shown that overexpression of RIG-I or MDA5 is inhibitory for HCV replication in Huh-7 cells.^34^ Additional studies are required to further define the mechanisms for V protein-mediated enhancement of HCV replication in primary culture.

Chimeric HCV genomes, such as those used in our study, enable the production of well-defined virus stocks for use in infection experiments. In contrast, HCV derived from patient plasma or tissues may be of variable infectious titer, and frequently complexed with virus-neutralizing antibody. Reports of successful infection of primary hepatocytes with such isolates are relatively rare, and the degree of replication achieved has varied widely between different culture systems.^6^-^11^ Few have reported sustained production of titratable infectious virus. Many factors may contribute to this variability, including the differentiation state of cultured hepatocytes and differences between HCV genomes in their relative dependence on cellular cofactors required for virus replication and spread.

Our results with HCVcc suggest that the hepatocyte innate response to infection may provide an additional barrier to productive replication in primary culture. Strategies aimed at dampening this response may be key to the further development of robust HCV culture systems based on infection or transfection of viral genomes.

## Abbreviations

2′CMA: 2′C-methyl adenosine
AAT: alpha-1 antitrypsin
AFP: alpha fetoprotein
CK: cytokeratin
CLDN1: claudin 1
Fluc: firefly luciferase
HCV: hepatitis C virus
HCVcc: cell culture-derived HCV
HDM: hepatocytes defined medium
HFLC: human fetal liver cells
HWB: hepatocyte wash buffer
IFN: interferon
JC1G: Jc1FLAG2p7-nsGluc2A
MV: measles virus
OCLN: occludin
PIV5: parainfluenza virus 5
PMV: paramyxovirus
PP: pseudoparticle
RLU: relative light units
SRB1: scavenger receptor B1
STAT: signal transducer and activation of transcription
TCID_50_: fifty percent tissue culture infectious doses
WEM: Williams'E medium
ZO1: zona occludens 1

## Supplementary material

Supporting Video 1: Firefly luciferase (Fluc)

Supporting Video 2: Parainfluenza virus 5 V protein (PIV5 V) or (C).

Supporting Video 3: Measles virus V protein (MV V).
