# Supplementary figures and images for: Expression of Paramyxovirus V Proteins Promotes Replication and Spread of Hepatitis C Virus in Cultures of Primary Human Fetal Liver Cells
Source: Hepatology. 2011 Dec 2;54(6):1901–12. doi: 10.1002/hep.24557 (PMC3233237; doi:10.1002/hep.24557)

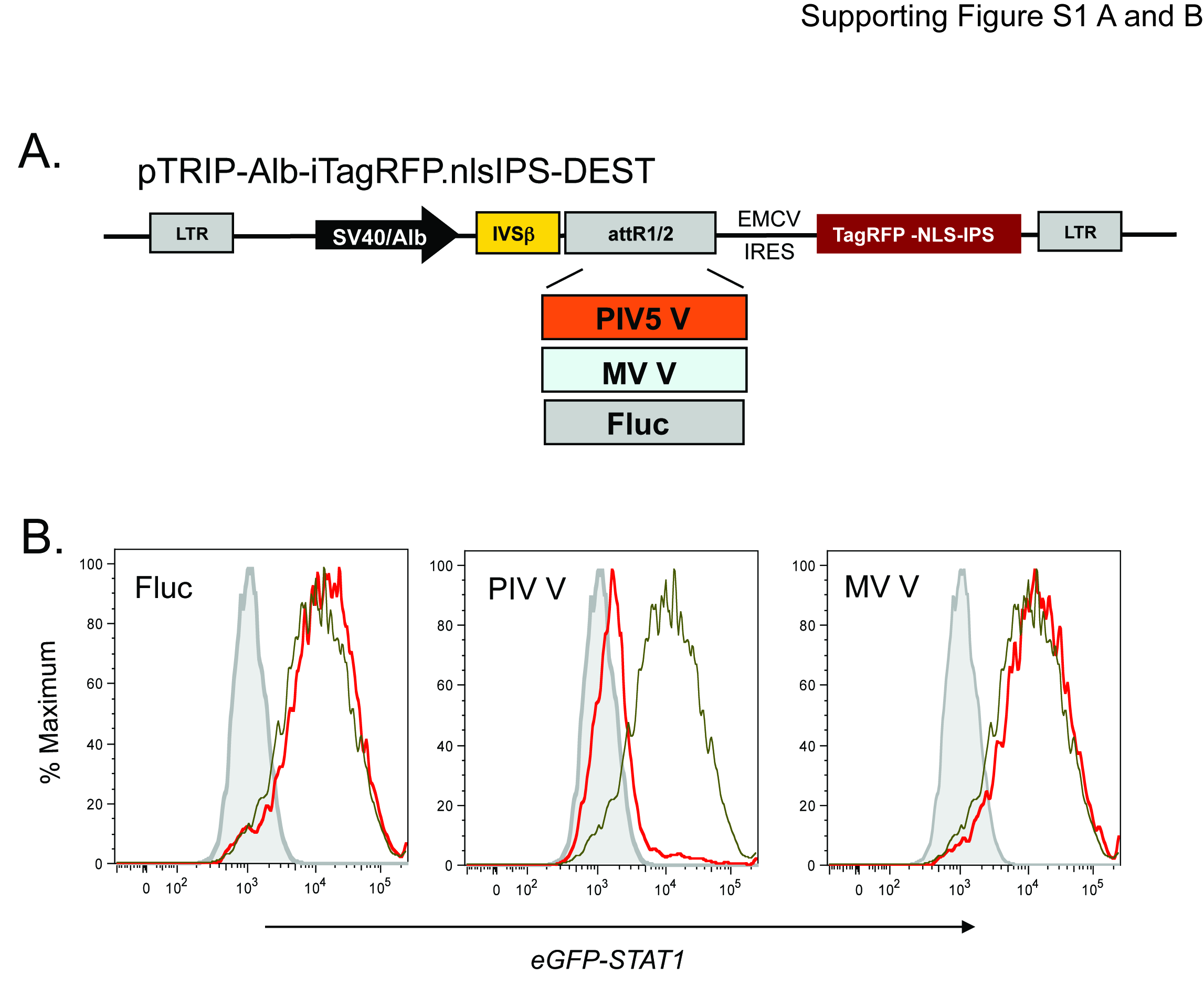

Supplement: Supplementary file 1 [file hep0054-1901-SD1.tif]

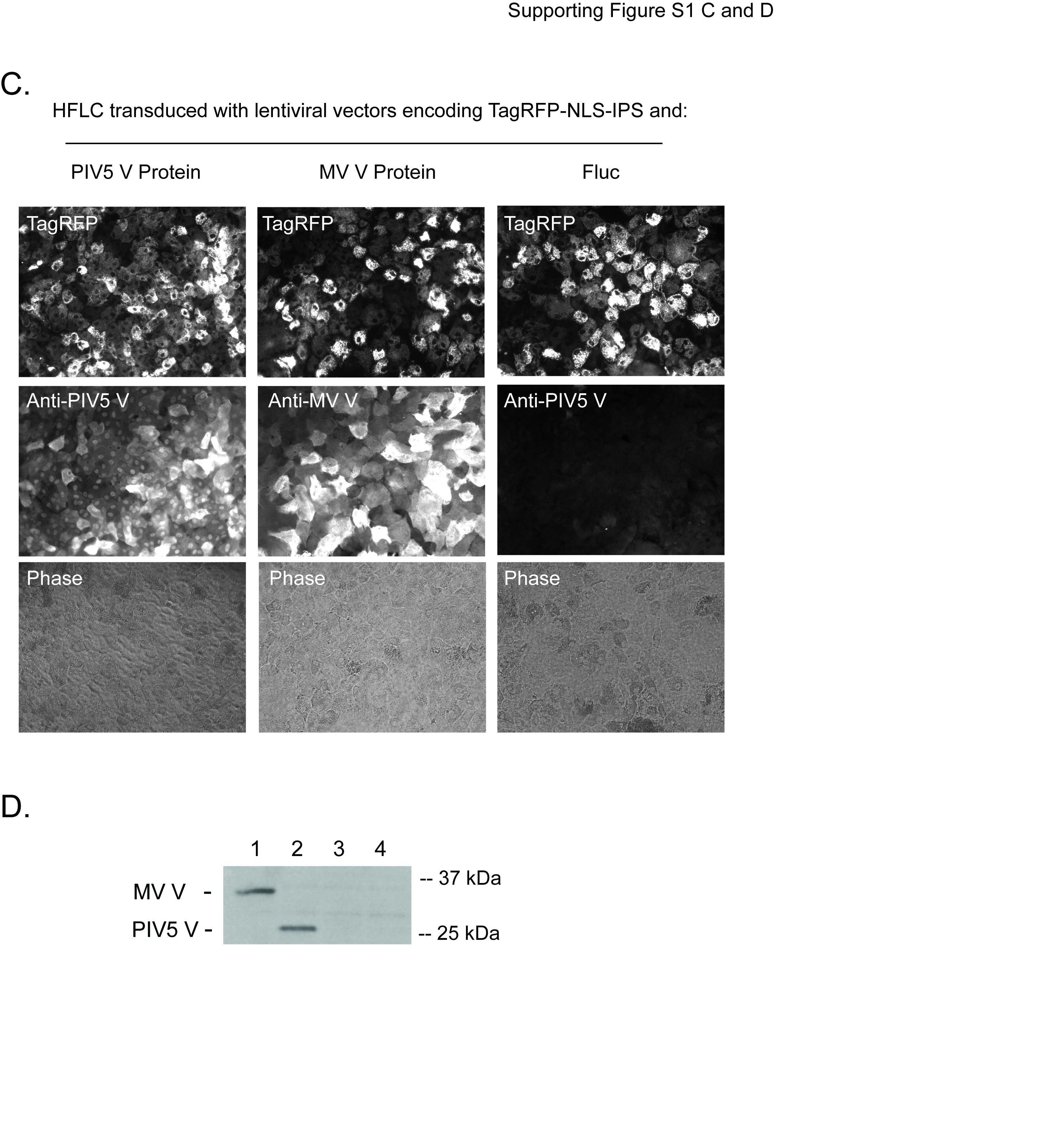

Supplement: Supplementary file 2 [file hep0054-1901-SD2.tif]

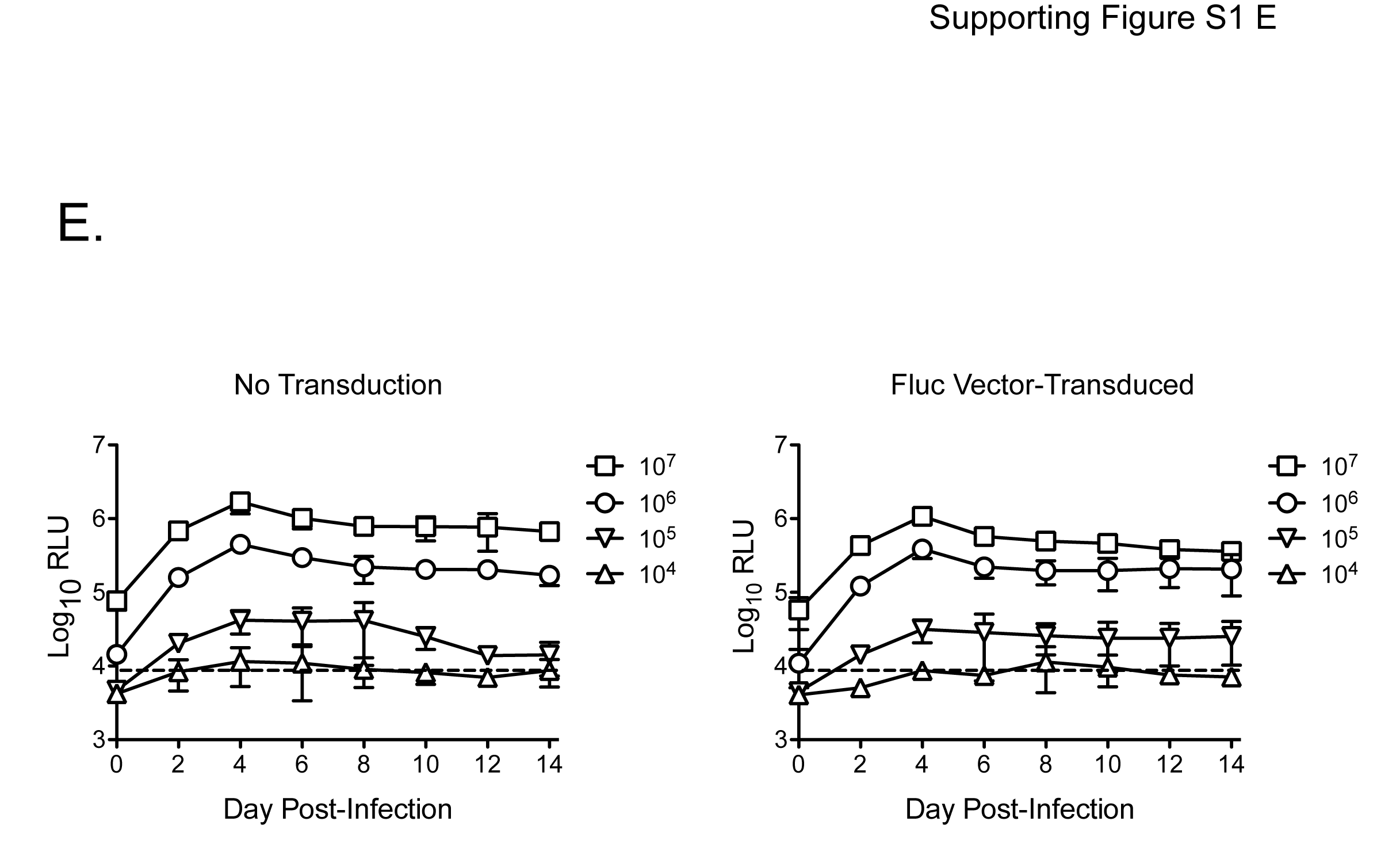

Supplement: Supplementary file 3 [file hep0054-1901-SD3.tif]

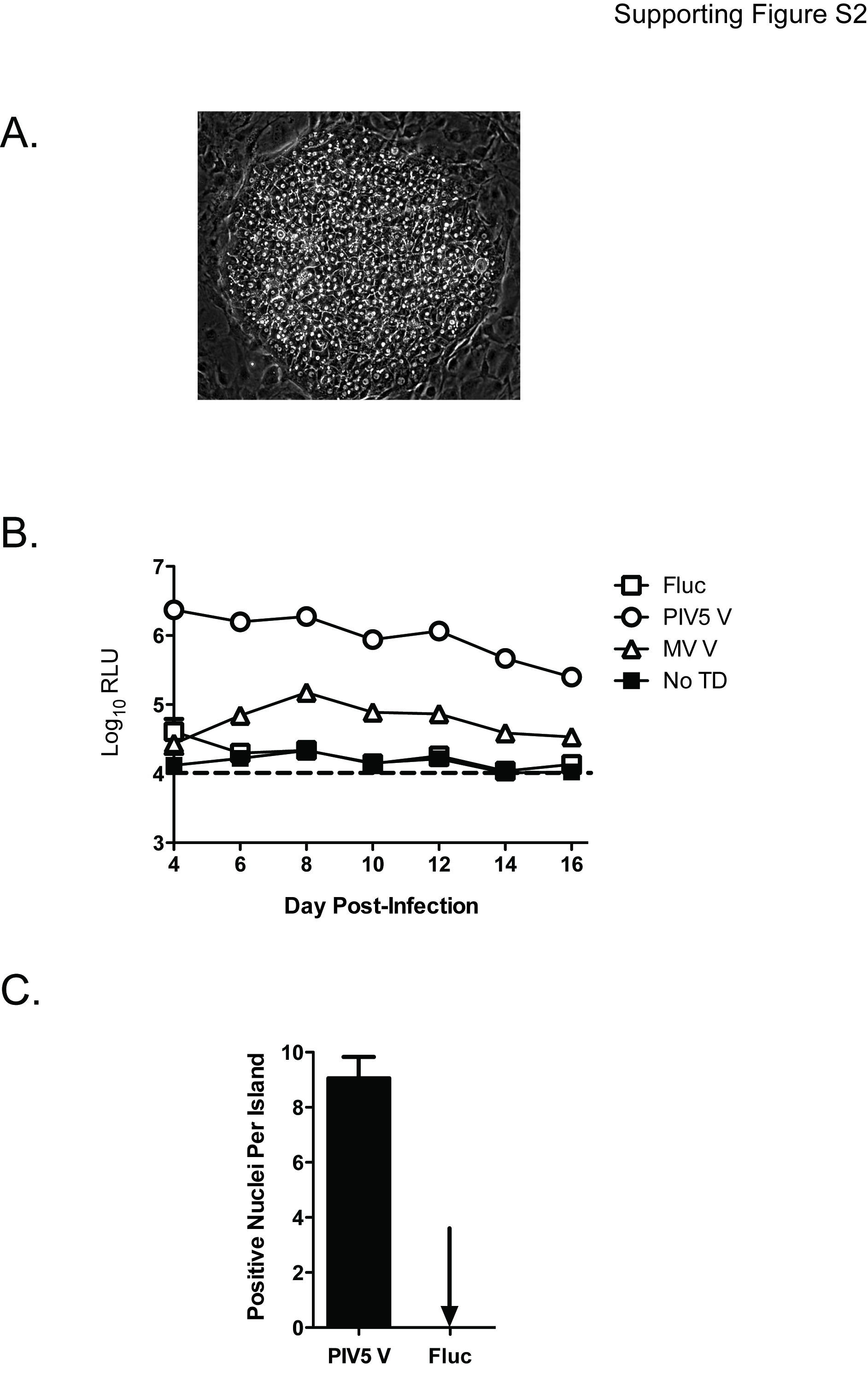

Supplement: Supplementary file 4 [file hep0054-1901-SD4.tif]

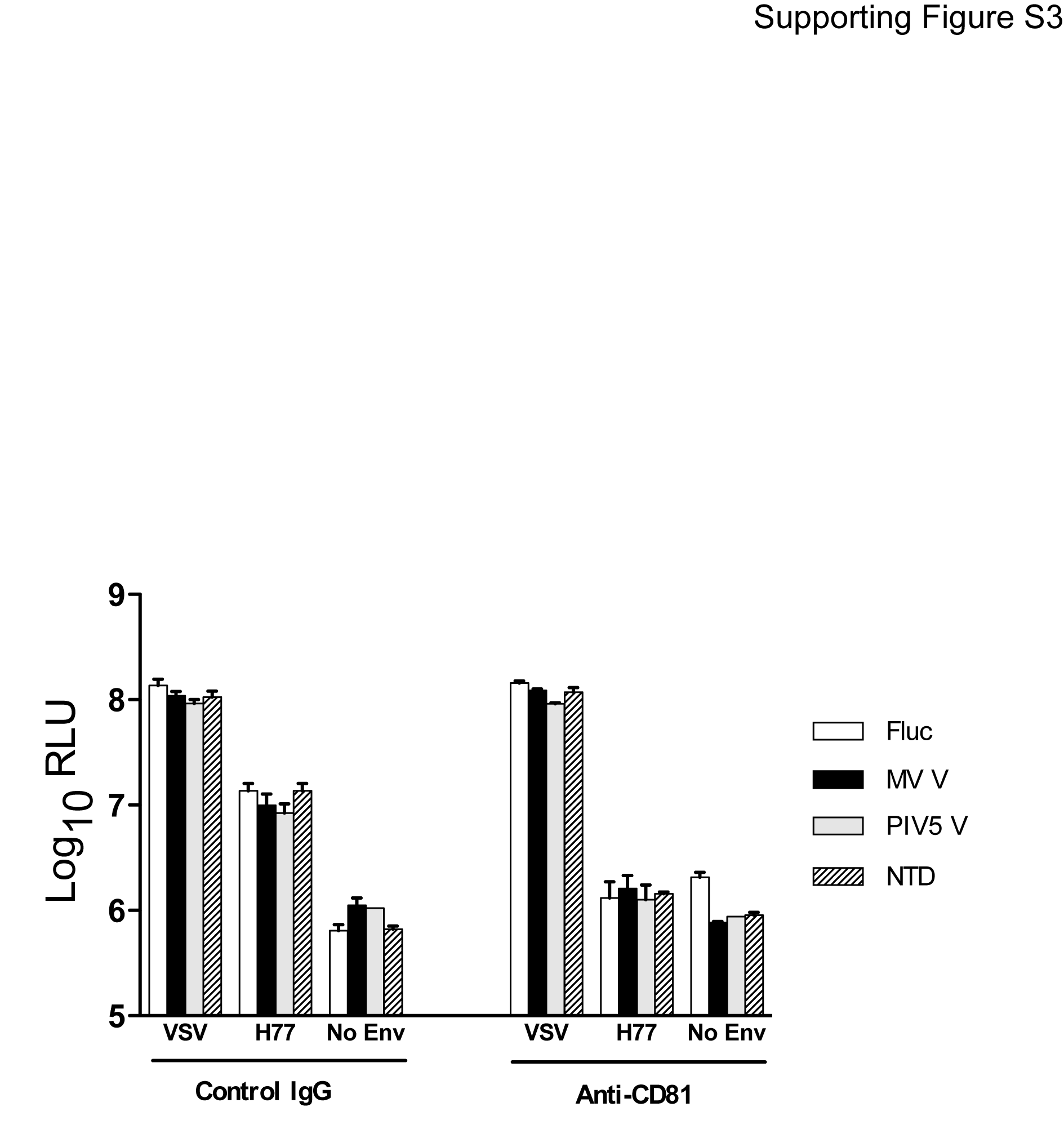

Supplement: Supplementary file 5 [file hep0054-1901-SD5.tif]
