# Supplementary material for: Expression of Paramyxovirus V Proteins Promotes Replication and Spread of Hepatitis C Virus in Cultures of Primary Human Fetal Liver Cells
Source: Hepatology. 2011 Dec 2;54(6):1901–12. doi: 10.1002/hep.24557 (PMC3233237; doi:10.1002/hep.24557)
Supplement: Supplementary file 6 [file hep0054-1901-SD6.doc]

**SUPPORTING METHODS**

**Construction of lentiviral vectors**

To construct lentiviral vectors allowing efficient expression of PMV V proteins or control proteins in hepatocytes, the CMV promoter in the destination vector pTRIP.CMV.IVSb.ires.TagRFP-DEST [34] was replaced with a hybrid SV40 human serum albumin promoter obtained by PCR from the plasmid pDrive-SV40-hAlb (Invivogen). To allow identification of HCV infected cells, TagRFP was replaced with the HCV-dependent fluorescence relocalization (HDFR) cassette, TagRFP-NLS-IPS [32], using NheI and SacII sites to create the destination vector pTrip.Alb.IVSb.ires.TagRFP-NLS-IPS-DEST (Supporting Figure 1A). Gateway cloning into the destination vector was achieved by constructing entry plasmids for each V protein and control protein sequence using the pENTR221 system (Invitrogen). Coding sequences for PIV5 and MV V proteins were obtained by PCR from expression plasmids generously provided by Dr. Curt Horvath. The sequence encoding the control protein firefly luciferase (Fluc) was PCR amplified from pGL41.2 (Promega).

**Immunofluorescence analysis (IFA)**

IFA was carried out as described [9] on washed HFLC cultures fixed with 1% paraformaldehyde. Primary antibodies were: rabbit anti-albumin (Dako, A0001; 1/500), goat anti-alpha-1 antitrypsin (AAT; Bethyl Laboratories, A80-122A; 1/500), rabbit anti-alpha fetoprotein (AFP; Dako, A0008; 1/500), mouse anti-cytokeratin 8 (CK8; Zymed, 18-01852, 1/250), mouse anti-cytokeratin 7 (CK7; Zymed, 18-0234; 1/250), mouse anti-vimentin (Invitrogen, 81052; 1/500), mouse anti-zona occludens 1 (ZO1; Zymed, 61-7300; 1/200), mouse anti-occludin (OCLN; Zymed, 33-1500; 1/250), and rabbit anti-claudin 1 (CLDN1; Zymed, 51-9000, 1/250). Rabbit antibodies to PIV5 and MV V proteins were the gift of Drs Curt Horvath and Roberto Cattaneo. AlexaFluor-labeled secondary antibodies were from Invitrogen.

**Immunoblot analysis**

Cell lysis, protein separation and blotting were performed as described [16]. Blots were probed with mouse anti-CD81 (BD Biosciences, 555675; 1/500), rabbit anti-scavenger receptor BI (SRBI; Abcam, ab396; 1/10,000), mouse anti-CLDN1 (Invitrogen 374900; 1/200) and mouse anti-OCLN (Invitrogen, 331500; 1/200). Horseradish peroxidase-conjugated secondary antibodies to rabbit or mouse IgG were from Jackson ImmunoResearch. Peroxidase-conjugated mouse anti-actin (Sigma, A3854; 1/10,000) was used to assess protein loading. Bound antibody was visualized using SuperSignal West Pico (Thermo Scientific).

**ELISA for human albumin**

Albumin in HFLC supernatants was measured by antigen capture with goat anti-human albumin (Bethyl Laboratories, A80-129A), and detection with mouse anti-human albumin (Abcam, ab399), biotin-conjugated goat anti-mouse IgG (Abcam, ab6788) and peroxidase-conjugated streptavidin (Zymed). Coating, blocking and assay development were performed as described [40]. Assays were calibrated using a standard curve prepared from dilutions of human albumin (Swiss Red Cross). Protein concentrations in unknown samples were calculated relative to protein standards using Softmax Pro software (Molecular Devices, Sunnyville CA).

**Culture, transduction and infection of primary adult hepatocytes**

Cryopreserved primary adult human hepatocytes obtained from CellzDirect lot Hu4151 (50 y/o, female Caucasian) were used to produce micropatterned co-cultures (MPCCs) with mouse J2-3T3 fibroblasts as described [9,41]. Hepatocytes were maintained in a DMEM with L-glutamine-based medium consisting of 1% ITS stock (BD Bioscences), 0.04 ug/ml dexamethasone (Sigma), 100 U/ml penicillin and 100 ug/ml streptomycin (Cellgro), 10% FBS (GIBCO), 15 mM HEPES buffer, and 7 ng/ml glucagon (Sigma). MPCCs) form a polarized layer of hepatocytes (Supporting Figure 2A) that retains hepatic phenotype for several weeks in culture [39]. MPCCs support HCV infection when dosed with high titer HCVcc (1x10^6^ TCID_50_) [11].

Adult hepatocytes were transduced 2 days post-plating by overnight incubation with PP stocks diluted 1:3 in medium supplemented as described for HFLC, then washed and fed. For HCVcc infection, cells were incubated with 1x10^5^ TCID_50_ virus diluted in ITS-based medium for 24 hr and subsequently washed 5 times and fed.

**SUPPORTING REFERENCES**

1. Nichols CN, Bernal I, Prince AM, Andrus L. Comparison of two different preparations of HIV immune globulin for efficiency of neutralization of HIV type 1 primary isolates. AIDS Res Hum Retroviruses 2002;18:49-56.
2. Khetani SR, Bhatia SN. Microscale culture of human liver cells for drug development. Nat Biotechnol 2008;26:120-126.

**LEGENDS TO SUPPORTING FIGURES**

**Supporting Figure S1: Construction and characterization of lentiviral vectors encoding V proteins or control protein Fluc and the TagRFP-NLS-IPS reporter. (A).** Destination vector pTRIP-Alb-iTagRFP.nlsIPS-DEST. Expression of V proteins or control protein Fluc is driven by a hybrid SV40/albumin promoter. Expression of TagRFP-NLS-IPS in the second cistron is driven by the encephalomyocarditis virus (ECMV) internal ribosome entry site (IRES). IVSβ, beta globin intron, attR1/2, Gateway cloning site. (**B**). The PIV5 V protein efficiently degrades STAT1. HuH7 cells stably expressing eGFP-tagged STAT1 were either left untransduced (green) or transduced (red) with each of the lentiviral vectors described above. Six days later, eGFP fluorescence was compared with that of the eGFP-negative parent cell line HuH7 (grey). (**C**). Expression of TagRFP-NLS-IPS and IFA for PIV5 or MV V proteins in HFLC. HFLC were transduced one day post-plating and fixed with 1% paraformaldehyde six days later. Cultures were stained with antibodies to either PIV5 V protein or MV V protein as indicated, and bound antibody was detected with AlexaFluor-488 conjugated antibody to rabbit IgG. Top row = visualization of the TagRFP-NLS-IPS reporter which is predominantly mitochondrially associated; Middle row = visualization of AF-488 fluorescence; Bottom row = phase contrast images of the same field. (**D**). Western blot of HFLC lysates (30 ug protein) five days-post-transduction with pseudoparticles encoding TagRFP-NLS-IPS and Lane: (1) MV V protein, (2) PIV5 V protein, (3) Fluc or (4) no transduction. The blot was stained sequentially with rabbit antibody to PIV5 and MV V protein. Migration of molecular size markers is indicated at the right of the panel. (**E**). Vector transduction does not affect the course of HCVcc replication in HFLC. HFLC (AECOM-052810) were either left untransduced, or transduced with lentiviral vector encoding the control protein Fluc, then infected with graded doses of JC1G as described in Figure 2B. Secretion of Gaussia luciferase was monitored for 2 weeks. For ease of comparison, results for the non-transduced cells shown in Figure 2B are shown sided by side with results obtained with control vector transduced HFLC. RLU, relative light units.

**Supporting Figure S2: V protein expression rescues abortive HCVcc infection in primary adult hepatocyte MPCCs.** (**A**). Phase image of an established culture of polarized, primary adult hepatocytes in MPCCs (B). Low-level infection of MPCCs with JC1G (1x10^5^ TCID_50_) is significantly enhanced upon expression of PIV5 and MV V proteins as measured by luciferase activity. (**C**). TagRFP-NLS-IPS reporter monitored 16 days post-infection demonstrates number of infectious events per island of micropatterned adult hepatocytes in PIV5 V and Fluc transduced cells. PIV5 V-transduced cultures contained significantly more nuclear translocation events than Fluc-transduced hepatocytes.

**Supporting Figure S3: Effect of PMV V-protein expression on HCV envelope-mediated PP entry in HFLC.** HFLC were either left untransduced (NTD) or transduced with pseudoparticles expressing Fluc, MV V protein, or PIV5 V protein. Five days post-transduction, the cells were tested for their ability to support entry of VSV-enveloped (VSV), HCV-enveloped (H77) or non-enveloped (No Env) PP carrying the Gaussia luciferase reporter [17]. Prior to their addition to HFLC, PP were incubated with 5 ug/ml controi IgG or anti-CD81 for 1 hr at room temperature. Entry was assessed by measurement of secreted Gaussia luciferase at 24, 72 and 96 hrs post-inoculation. Results from 96 hrs are shown.
